# Portal vein hemodynamics measured by 4D flow MRI in predicting minimal hepatic encephalopathy in chronic hepatic schistosomiasis patients

**DOI:** 10.3389/fmed.2025.1681210

**Published:** 2025-10-16

**Authors:** Xue-Fei Liu, Ke-Ying Wang, Hai-Feng Shi, Ying Li, Xin Li

**Affiliations:** Department of Radiology, Jinshan Hospital of Fudan University, Shanghai, China

**Keywords:** minimal hepatic encephalopathy, chronic hepatic schistosomiasis, portal vein hemodynamics, 4D flow MRI, portal hypertension

## Abstract

**Background:**

Minimal hepatic encephalopathy (MHE) is a subclinical neuropsychological disorder frequently observed in chronic hepatic schistosomiasis (CHS) patients. Early diagnosis is challenging due to the subtle and nonspecific nature of MHE symptoms. Advances in magnetic resonance imaging (MRI), specifically 4D flow MRI, allow for non-invasive evaluation of portal vein hemodynamic parameters, offering a potential tool for diagnosing MHE.

**Objective:**

To evaluate the application of portal vein hemodynamic parameters measured by 4D flow MRI in diagnosing MHE in CHS patients.

**Methods:**

This prospective observational study included 118 CHS patients, divided into MHE (*n* = 52) and non-MHE (*n* = 66) groups. Portal vein hemodynamic parameters, including peak velocity, mean velocity, flow volume, and vessel area, were measured using 4D flow MRI. Correlation analyses were used to assess the relationship between these parameters and MHE. Diagnostic performances of portal vein hemodynamic parameters in MHE were also evaluated.

**Results:**

Patients with MHE exhibited significantly lower portal peak velocity (*p* < 0.001) and mean velocity (*p* < 0.001). Portal area (*p* < 0.001) was higher in MHE patients. Correlation analysis revealed significant associations between MHE and portal peak velocity (*r* = −0.389), mean velocity (*r* = −0.566), and area (*r* = 0.435). The diagnostic performance of portal peak velocity, mean velocity, and area in MHE demonstrated AUC of 0.70 (95% CI: 0.61–0.79) with specificity, sensitivity, negative predictive value (NPV) and positive predictive value (PPV) of 0.92, 0.47, 0.58, and 0.89, respectively; AUC of 0.82 (95% CI: 0.74–0.89) with specificity, sensitivity, NPV and PPV of 0.98, 0.64, 0.68, and 0.98, respectively; AUC of 0.74 (95% CI: 0.65–0.83) with specificity, sensitivity, NPV and PPV of 0.94, 0.46, 0.69, and 0.86, respectively.

**Conclusion:**

4D flow MRI-derived portal vein hemodynamic parameters, particularly portal mean velocity, are significantly associated with MHE in CHS patients. These findings suggest that non-invasive imaging can provide insights for diagnosis and management of MHE, potentially improving clinical outcomes.

## Introduction

Chronic hepatic schistosomiasis (CHS) remains a significant global health burden, and it contributes to substantial morbidity and mortality. The most severe complication of CHS is portal hypertension and portosystemic shunts (1, 2). These pathological changes can lead to clinical manifestations, including minimal hepatic encephalopathy (MHE) (3). MHE, a subclinical neuropsychological disorder, impacts cognitive and motor functions, often reducing patients’ quality of life and productivity (4). Despite its prevalence, early diagnosis of MHE is frequently challenging due to its subtle and nonspecific symptoms (5).

Portal hypertension plays a central role in the development of MHE by promoting the formation of portosystemic shunts and the systemic circulation of gut-derived toxins (1, 2). Advanced neuroimaging techniques have revealed dynamic and static brain fluctuation changes in MHE patients, providing new insights into the neural basis of cognitive dysfunction (6, 7). The gut-liver-brain axis has also emerged as a critical pathway in MHE pathogenesis, with studies demonstrating that gut microbiota dysbiosis subsequently leads to neuroinflammation in the brain (8). Despite these advances, the heterogeneity of underlying liver diseases and the subtle nature of MHE symptoms continue to pose diagnostic challenges, particularly in populations with CHS where portal hypertension patterns may differ from those seen in viral or alcoholic liver disease. Identifying reliable, non-invasive predictors of MHE in CHS patients is essential to enable timely interventions and prevent progression to overt hepatic encephalopathy. However, traditional diagnostic methods for MHE often lack specificity or are influenced by patient compliance and external factors (9–11).

Recent studies have underscored the utility of non-invasive imaging techniques in evaluating portal vein hemodynamics and predicting complications of portal hypertension. A study highlighted the relationship between altered portal vein hemodynamics and cognitive dysfunction in patients with portal hypertension (12). Previous studies also demonstrated that reduced portal velocity correlates strongly with the risk of hepatic encephalopathy in patients with cirrhosis (13). And the hepatofugal flow within the portal vein has been confirmed to be the origin of the shunt and the cause of the clinical manifestations of hepatic encephalopathy (14). These findings suggest that portal vein hemodynamic parameters can serve as a reliable marker for MHE. However, few studies have explored this relationship in the context of CHS.

Advances in MRI technology, particularly 4D flow MRI, have enabled comprehensive and non-invasive assessment of blood flow in the portal venous system (28). It allows for accurate quantification of portal vein hemodynamic parameters, such as blood flow velocity, volume, and vessel area, in the portal vein and its branches. By offering detailed insights into portal vein hemodynamics, 4D flow MRI can help detect early alterations associated with portal hypertension and its complications (6, 7).

We assumed that the application of 4D flow MRI-derived portal vein hemodynamic parameters could be used as a diagnostic tool for MHE in CHS patients. This study evaluated the relationship between portal vein hemodynamic parameters and the risk of MHE and discusses the potential of integrating 4D flow MRI into clinical practice to improve outcomes in this vulnerable patient group.

## Methods

### Ethical considerations

This study adhered to the ethical guidelines outlined in the Declaration of Helsinki. Ethical approval was obtained from the institutional review board of Jinshan Hospital (JIEC2023-S66). Written informed consent was obtained from all participants prior to enrollment. Participants were informed of the study’s objectives, procedures, and potential risks and benefits. Confidentiality was maintained by anonymizing patient data and securing the database with restricted access.

### Study design and sample size calculation

This study was designed as a prospective observational investigation to explore the association between portal vein hemodynamic parameters, as measured by 4D flow MRI, and the risk of MHE in CHS patients. The sample size was estimated by post-hoc power analysis based on the ability to detect a significant difference in portal vein flow velocity between MHE and non-MHE with the expected minimum difference of 3 cm/s. Assuming a two-tailed test with a significance level (*α*) of 0.05 and power (1 − β) of 0.80, the required sample size per group was calculated to be approximately 50 participants.

### Participants

Between August 15, 2023 and July 15, 2024, the study enrolled 139 eligible patients diagnosed with CHS consecutively. Participants were recruited through referrals from the local hepatology clinics and department of gastroenterology. Participants were stratified into two groups based on the presence or absence of MHE. Baseline clinical, demographic, laboratory, and imaging data were collected for all participants.

### Inclusion and exclusion criteria

The inclusion criteria were as follows: (1) A confirmed diagnosis of CHS; (2) Willingness to provide informed consent and undergo all required assessments, including MRI and psychometric testing.

The exclusion criteria were as follows: (1) History of recent (within the last 6 months) gastrointestinal bleeding or other acute complications; (2) Comorbidities such as neurodegenerative diseases, significant head trauma, or psychiatric disorders or presence of overt hepatic encephalopathy; (3) Contraindications to MRI; (4) Poor-quality MRI images.

### Diagnosis of chronic hepatic schistosomiasis

The diagnosis of CHS was based on a combination of clinical, laboratory, and imaging criteria. Clinical history included evidence of exposure to Schistosoma-endemic regions or prior treatment for schistosomiasis. Laboratory confirmation was performed using serological tests to detect antibodies against Schistosoma species. Imaging findings characteristic of CHS, such as periportal fibrosis, liver calcifications, splenomegaly, or collateral vessel formation, were assessed using abdominal ultrasound or CT.

### MHE assessment

The diagnosis of MHE was based on a combination of the Number Connection Test-A (NCT-A) and Digit Symbol Test (DST). In the NCT-A, patients rapidly connected numbered circles (1–25) while being timed; The DST required transcribing symbols paired with digits (1–9) under a 90-s deadline. With the criteria reported previously, the combining NCT-A and DST was able to diagnose MHE with a sensitivity of 76.9% and a specificity of 96.3% in the Chinese population (10, 11).

### Laboratory tests

Laboratory tests were performed on the same day as MRI scanning. All laboratory tests were conducted to evaluate liver function including alanine aminotransferase (ALT), aspartate aminotransferase (AST), total bilirubin (TB), albumin (ALB), prothrombin time (PT), international normalized ratio (INR), and platelet count (PLT).

### Magnetic resonance imaging protocol

MRI examinations were conducted using a 3.0 Tesla Siemens system (uMRI 780, United Imaging, Shanghai) equipped with advanced 4D flow imaging capabilities to assess portal vein hemodynamics. 4D flow MRI sequence allows for high-resolution, time-resolved visualization of blood flow, enabling precise measurement of velocities and flow volumes. Respiratory gating using navigator echoes acquisition with the respiratory cycle, and cardiac gating using ECG triggering for consistent cardiac phase timing were used to minimize motion artifacts. Imaging parameters included a repetition time of 44.16 ms, an echo time of 3.25 ms, and a flip angle of 20°. The field of view was set to 340 × 340 mm.

Regions of interest (ROIs) were carefully delineated on the cross-sectional images of the main portal vein and its primary branches. Each ROI was placed approximately 1 cm from the bifurcation of the portal vein to ensure consistency across measurements. Portal vein hemodynamic parameters extracted included peak velocity (maximum blood flow velocity in cm/s), mean velocity (average blood flow velocity in cm/s over the cardiac cycle), flow volume (total volume of blood flow in mL/s), and area (cross-sectional area of the portal vein in cm^2^). For each patient, three ROIs were drawn by one radiologist (Xin Li with 18 years experience in abdominal radiology blind to the MHE status), and the mean values derived from them were utilized as the final values.

### Statistical analysis

All analyses were conducted using R software (version 4.5.0). Continuous variables were expressed as mean ± standard deviation and tested for normality. Depending on the distribution, comparisons between groups were conducted using Student’s *t*-test (for normally distributed data) or the Mann–Whitney U test (for non-normally distributed data). Categorical variables were compared using Chi-square tests. To identify relationships between MRI-derived portal vein hemodynamic parameters and MHE, correlation analyses were performed using Pearson or Spearman correlation coefficients, depending on data distribution. Receiver operating characteristic (ROC) curve analysis was employed to assess the predictive accuracy of the logistic regression model, with the area under the curve (AUC) used as a measure of diagnostic performance. Statistical significance was set at a *p*-value of <0.05.

## Results

### Demographic and clinical characteristics

**Figure 1:**
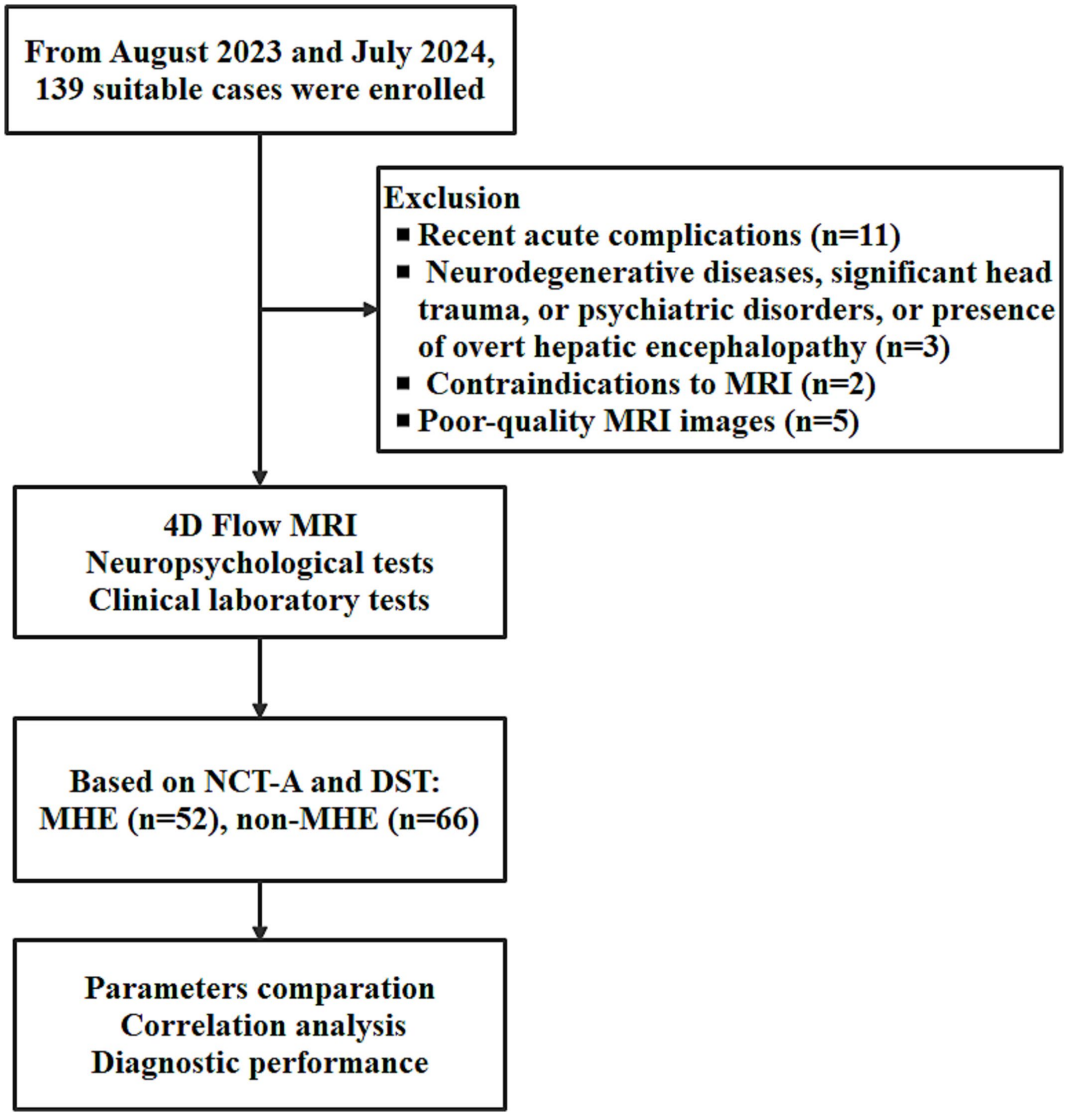
The work flow of this study. NCT-A, number connection test-A; DST, digit symbol test; MHE, minimal hepatic encephalopathy.

The work flow of this study is shown in Figure 1. After excluding 21 cases according to the exclusion criteria (Figure 1). The study included 118 CHS patients, divided into two groups: 52 patients with MHE and 66 patients without MHE (non-MHE). Gender distribution was comparable between the groups, with females comprising 36.5% in the MHE group and 43.9% in the non-MHE group (*p* = 0.533). The mean age was also similar between the groups, with 68 ± 8.5 in the MHE group and 67 ± 11.1 in the non-MHE group (*p* = 0.355). No difference in education level was found between the groups (*p* = 1.000). The clinical and laboratory characteristics and portal vein hemodynamic parameters in MHE and non-MHE cases are shown in Table 1 and Supplementary Table 2.

**Table 1:** The clinical and laboratory characteristics and portal vein hemodynamic parameters in MHE and non-MHE cases.

Parameters	MHE (*N* = 52)	Non-MHE (*N* = 66)	*p*-value
Gender			0.533
Female	19 (36.5%)	29 (43.9%)	
Male	33 (63.5%)	37 (56.1%)	
Age (y)	68 ± 8.5	67 ± 11.1	0.355
Education level			1.000
Above primary school	4 (7.7%)	6 (9.1%)	
Below primary school	48 (92.3%)	60 (90.9%)	
NCT-A	66 ± 11.4	37 ± 8.4	<0.001
DST	28 ± 8.1	45 ± 14.5	<0.001
AST (U/L)	46 ± 27.8	33 ± 18.8	0.004
ALT (U/L)	54 ± 24.3	49 ± 27.3	0.272
TB (μmol/L)	32.2 ± 12.2	26.7 ± 10.7	0.011
ALB (g/L)	30 ± 5.3	29 ± 5.5	0.849
PT (s)	13.6 ± 3.26	12.8 ± 3.30	0.220
INR	1.23 ± 0.13	1.24 ± 0.13	0.831
PLT (10^9^/L)	95 ± 58.6	111 ± 53.4	0.271
Portal peak velocity (cm/s)	17.6 ± 2.48	20.7 ± 4.46	<0.001
Portal mean velocity (cm/s)	11.6 ± 1.56	15.4 ± 3.45	<0.001
Portal flow (mL/s)	13.5 ± 4.31	12.7 ± 5.42	0.382
Portal area (cm^2^)	1.15 ± 0.29	0.85 ± 0.32	<0.001
Left branch peak velocity (cm/s)	12.0 ± 4.05	12.6 ± 3.29	0.401
Left branch mean velocity (cm/s)	7.9 ± 3.07	8.1 ± 2.83	0.736
Left branch flow (mL/s)	3.1 ± 1.43	2.5 ± 1.94	0.074
Left branch area (cm^2^)	0.38 ± 0.23	0.33 ± 0.17	0.261
Right branch peak velocity (cm/s)	15.1 ± 1.65	15.3 ± 3.33	0.621
Right branch mean velocity (cm/s)	9.6 ± 1.39	9.8 ± 3.73	0.697
Right branch flow (mL/s)	7.0 ± 2.65	6.2 ± 2.98	0.128
Right branch area (cm^2^)	0.67 ± 0.27	0.67 ± 0.38	0.997

ALB, albumin; ALT, alanine aminotransferase; AST, aspartate aminotransferase; DST, digit symbol test; INR, international normalized ratio; MHE, minimal hepatic encephalopathy; NCT-A, number connection test-A; PLT, platelet count; PT, prothrombin time; TB, total bilirubin.

### NCT-A and DST

NCT-A were markedly elevated in the MHE group compared to the non-MHE group (*p* < 0.001). DST, however, was decreased in the MHE group compared to the non-MHE group (*p* < 0.001).

### Liver function and biochemical markers

Patients with MHE exhibited significantly higher AST (*p* = 0.004) and TB (*p* = 0.011) compared to the non-MHE group. While ALT (*p* = 0.272), PT (*p* = 0.220), INR (*p* = 0.831), and PLT (*p* = 0.271) showed no differences between the groups.

### Portal vein hemodynamic parameters

**Figure 2:**
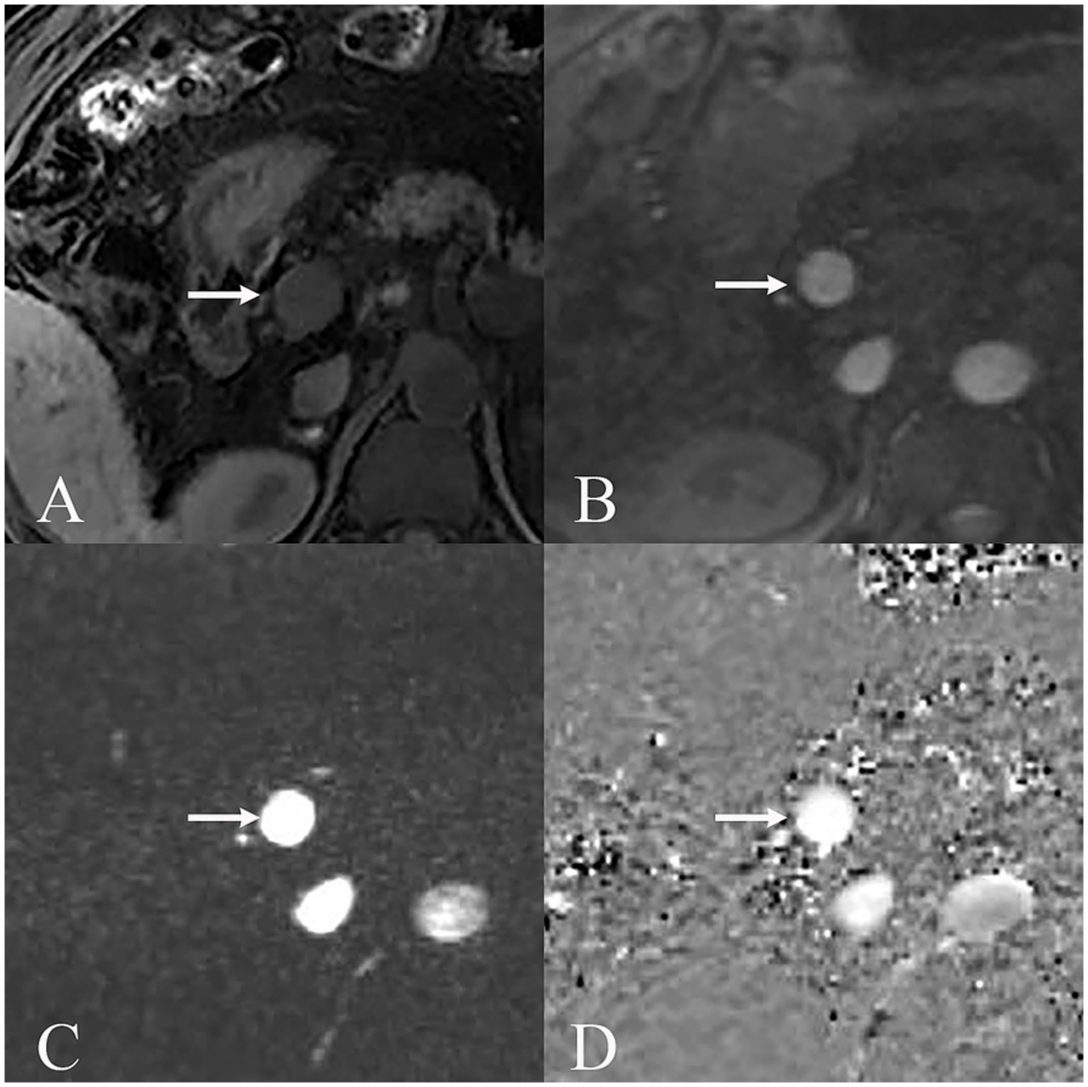
MRI images displaying portal vein (arrow) under various sequences used for MRI 4D Flow. **(A)** Non-enhanced T1WI showed the main portal vein and surrounding anatomical structures. **(B)** Scout image displays the portal vein. **(C)** Magnitude image demonstrates the portal vein with good vessel contrast and definition for flow quantification. **(D)** Phase contrast image shows flow-related signal intensity changes within the portal vein, where signal intensity corresponds to velocity encoding in the through-plane direction.

Patients with MHE demonstrated significantly lower portal peak and mean velocities compared with the non-MHE group (peak velocity: *p* < 0.001; mean velocity: *p* < 0.001). Portal area was also larger in the MHE group compared with the non-MHE group (*p* < 0.001). No significant differences in flow volume was observed between the groups (*p* = 0.382). A scanning case of 4D flow MRI is shown in Figure 2.

### Secondary hemodynamic parameters

In secondary portal branches (left and right), no significant differences in peak velocity, mean velocity, flow volume, or area were observed between the groups (all *p* > 0.05).

### Multivariable adjustment and correlation analyses

All risk factors (including AST, TB, portal peak velocity, portal mean velocity, portal area, left portal branch flow) with a *p*-value lower than 0.05 in the univariate logistic regressions were included in the multivariate logistic regression. Multivariable logistic regression showed that higher portal mean velocity (*p* < 0.001) is strongly associated with increased odds of MHE, while larger portal area decreases these odds (*p* = 0.016). portal peak velocity shows a borderline significant positive association (*p* = 0.064), while demographic factors (gender, age) and left portal branch flow show no significant associations with MHE. The result of the multivariable logistic regression is shown in Supplementary Table 1.

**Figure 3:**
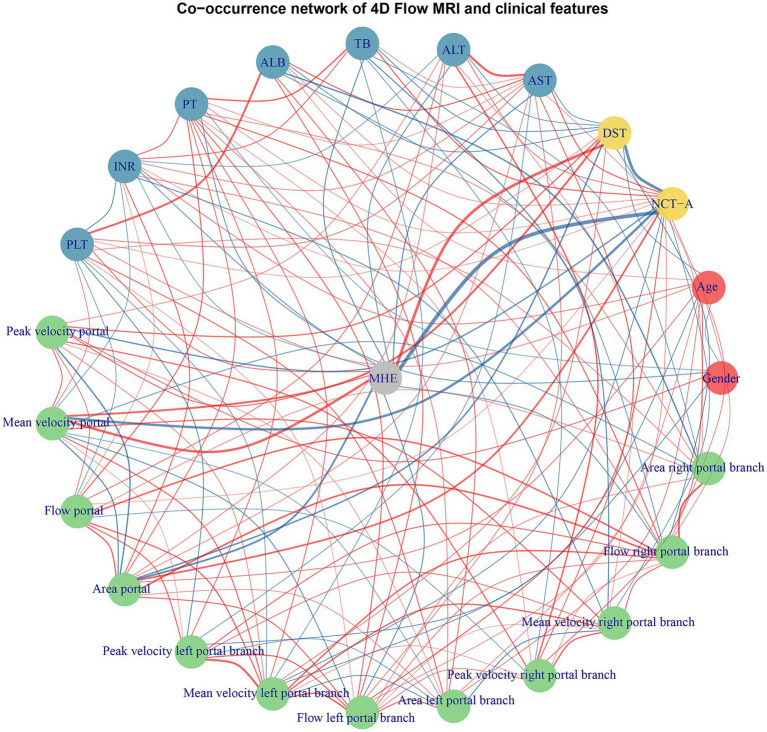
Co-occurrence network analysis of portal vein hemodynamic parameters and clinical features. The network visualization displays the interrelationships between portal vein hemodynamic parameters and clinical variables. Nodes represent different variable categories. Red edges indicate positive correlations while blue edges represent negative correlations between variables with the thickness of the line displaying the correlation coefficient value. ALB, albumin; ALT, alanine aminotransferase; AST, aspartate aminotransferase; INR, international normalized ratio; PLT, platelet count; PT, prothrombin time; TB, total bilirubin.

Correlation analyses were conducted to examine the relationship between MHE and key portal vein hemodynamic parameters. Significant negative correlations were observed between MHE and both portal peak and mean velocity. Specifically, the correlation between MHE and portal peak velocity was −0.389 (*p* < 0.001), with a 95% confidence interval (CI) of −0.533 to −0.225. Similarly, portal mean velocity showed a stronger negative correlation of −0.566 (*p* < 0.001), with a 95% CI of −0.677 to −0.429. In contrast, portal area showed positive correlations with MHE, which demonstrated a correlation coefficient of 0.435 (*p* < 0.001), with a 95% CI of 0.276 to 0.571 (Figure 3).

### Diagnostic performance of portal vein hemodynamic parameters in MHE

**Figure 4:**
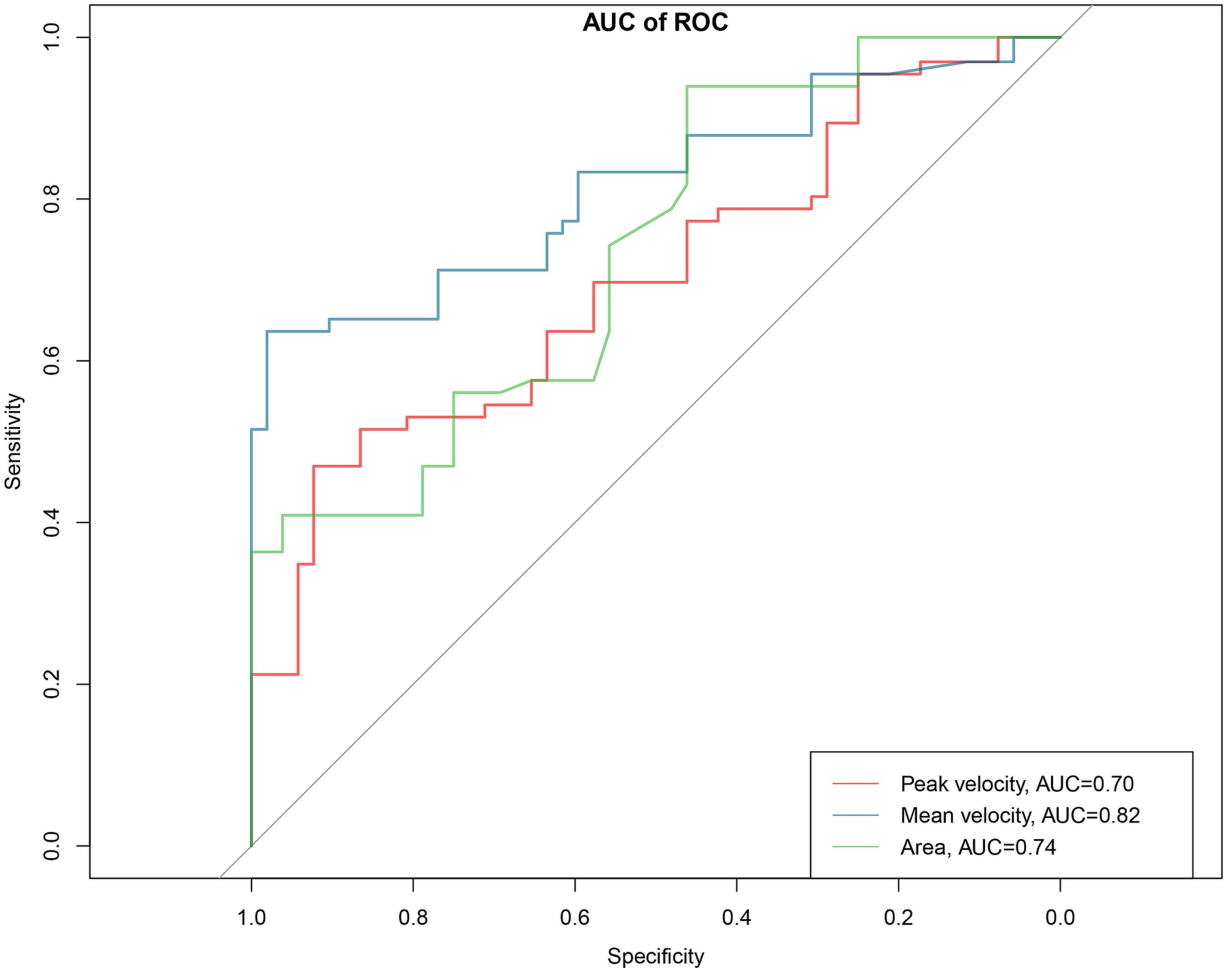
Area under the receiver operating characteristic (ROC) curve (AUC) analysis for portal vein hemodynamic parameters in diagnostic performance of MHE. The red, blue and green curve represents peak velocity, mean velocity and portal area.

The diagnostic performance of portal vein hemodynamic parameters in MHE is shown in Table 2 and Figure 4. Portal peak velocity demonstrated diagnostic performance with AUC of 0.70 (95% CI: 0.61–0.79) with specificity, sensitivity, negative predictive value (NPV), positive predictive value (PPV), positive likelihood ratio (LR+), and negative likelihood ratio (LR−) of 0.92, 0.47, 0.58, 0.89, 5.88, and 0.58, respectively. Portal mean velocity demonstrated diagnostic performance with AUC of 0.82 (95% CI: 0.74–0.89) with specificity, sensitivity, NPV, PPV, LR+, and LR− of 0.98, 0.64, 0.68, 0.98, 32.00, and 0.37, respectively. The portal area demonstrated diagnostic performance with AUC of 0.74 (95% CI: 0.65–0.83) with specificity, sensitivity, NPV, PPV, LR+, and LR− of 0.94, 0.46, 0.69, 0.86, 8.46, and 0.57, respectively.

**Table 2:** The diagnostic performance of portal vein hemodynamic parameters in MHE.

Parameters	AUC	95%CI	SPE	SEN	NPV	PPV
Portal peak velocity	0.70	0.61–0.79	0.92	0.47	0.58	0.89
Portal mean velocity	0.82	0.74–0.89	0.98	0.64	0.68	0.98
Portal area	0.74	0.65–0.83	0.94	0.46	0.69	0.86

AUC, area under the curve; CI, confidence interval; SEN, sensitivity; SPE, specificity; NPV, negative predictive value; PPV, positive predictive value.

## Discussion

This study highlights the potential of 4D flow MRI to assess portal vein hemodynamics as a non-invasive diagnostic tool for MHE in CHS patients. The findings showed lower portal peak and mean velocities but increased portal area in patients with MHE and CHS.

The primary cause of MHE in patients with liver cirrhosis is due to impaired liver function and subsequent elevated blood ammonia levels. In contrast, the main reason for MHE occurrence in patients with CHS is due to the portosystemic shunts resulting from portal hypertension (15). Due to the preserved liver synthetic function, the CHS patients usually have normal blood ammonia (10, 11). Clinical and animal studies have confirmed that the deposition of toxic substances in the brain, which are caused by portosystemic shunts and particularly involve manganese bypassing liver detoxification, is the principal cause of MHE development in CHS patients (1, 2, 15).

Chronic liver disease with portal hypertension, such as nonalcoholic fatty liver disease, fibrosis, and cirrhosis, accompanied by alterations in the hemodynamics of portal vein (16). Studies have highlighted the diagnostic potential of 4D flow MRI in evaluating portal vein hemodynamics and predicting complications of portal hypertension. 4D Flow MRI also reflects physiological hemodynamics for the diagnosis and management of portosystemic shunts (17). Previous studies showed that combined indices of portal vein hemodynamic parameters could effectively predict the occurrence of varices in cirrhotic patients (18). Another study showed 4D flow MRI could be used as an independent predictor for high-risk varices in cirrhotic patients with high sensitivity 100% (19). This study underscores the utility of 4D flow MRI as a non-invasive diagnostic tool of MHE. The result is in accordance with previous studies. Li et al. reported that measurement of hemodynamic changes has certain clinical significance for predicting hepatic encaphalopahty occurrence (20).

Previous studies showed that lower portal peak velocity in patients with hepatic autoinflammatory disease with the existence of portal hypertension (21). Another study demonstrated that elevated portal pressure and decreased portal velocities correlate in cirrhotic patients with portal hypertension (22). The significant reduction in portal peak and mean velocity in the MHE group underscores the association between altered portal vein hemodynamics and cognitive dysfunction. However, the results showed that the correlation between portal peak velocity and MHE was not as good as portal mean velocity, possibly because portal peak velocity is easily affected by respiration and heart rate (23).

The compensatory increase in portal area observed in MHE patients suggests vascular remodeling in response to chronic portal hypertension. These structural changes are consistent with findings from studies on upper gastrointestinal bleeding risk in chronic hepatic disease, where vessel distensibility mitigates elevated pressure but impairs portal vein hemodynamics (24). Additionally, studies have identified altered portal vein hemodynamic parameters as critical predictors of neurocognitive dysfunction, further linking vascular remodeling to MHE development (12). As the portal vein to aorta ratio is also reported useful in assessing the clinical significance of extrahepatic portosystemic shunts (25), which is associated with hepatic encephalopathy (26) and the main cause of MHE in CHS patients (15). While peak velocity and portal area demonstrate high specificities, the poor sensitivities make them more suitable as definitive diagnostic tests rather than screening tools. The peak velocity and portal area should be used in combination with other parameters or as part of a composite diagnostic algorithm rather than as isolated criteria, helping clinicians understand the appropriate clinical context for these hemodynamic parameters.

Given its modest sensitivity and technical challenges, 4D flow MRI may be better suited as a confirmatory test rather than a screening tool. When psychometric testing is equivocal or unreliable 4D flow MRI could be used to detect early MHE, which could potentially prevent progression to overt hepatic encephalopathy and reduce hospitalizations. However, compared to psychometric testing, the high costs of 4D flow MRI make the cost-effectiveness ratio likely unfavorable in resource-limited schistosomiasis-endemic regions. Therefore, 4D flow MRI should be integrated as part of a multimodal diagnostic approach rather than a standalone screening test. The integration of 4D flow MRI parameters with complementary clinical, laboratory, and imaging biomarkers might significantly enhance diagnostic accuracy for MHE, which could create more robust diagnostic algorithms with improved sensitivity and specificity. Furthermore, future work requires improved spatial–temporal resolution, enhanced motion correction algorithms, optimized low-velocity flow detection sequences, standardized protocols, and prospective health economic studies to determine its optimal clinical role and cost-effectiveness before routine clinical practice.

This study has several important limitations. The cross-sectional, single-center design with a relatively small sample size limits generalizability and prevents establishment of causal relationships or assessment of predictive utility over time. We did not systematically account for potential confounding factors including medications, comorbidities, or transient hemodynamic fluctuations. Additionally, inherent 4D flow MRI limitations including acquisition artifacts, post-processing variations, and operator-dependent measurements could affect reproducibility. Future research should focus on larger, multi-center, longitudinal studies to validate these findings across diverse patient populations and explore applications in other etiologies of portal hypertension. Additionally, comprehensive MHE diagnosis typically includes additional assessments such as psychometric hepatic encephalopathy score, critical flicker frequency, or neurophysiological tests, and our simplified approach may have missed some cases or caused misclassification compared to more comprehensive diagnostic batteries. Furthermore, this study’s focus was solely on main portal vein hemodynamics, rather than quantifying portosystemic shunt flow. Since portosystemic shunts are more directly linked to MHE development pathophysiology. The omission of comprehensive shunt assessment across collateral pathways limits the study’s ability to investigate the primary hemodynamic drivers of MHE, despite the technical feasibility demonstrated in other contexts such as variceal bleeding risk stratification (27).

## Conclusion

This study demonstrates the potential of 4D flow MRI to identify portal vein hemodynamic alterations associated with MHE in CHS patients. By providing a non-invasive assessment of portal vein hemodynamics, this technique offers a tool for the detection and management of MHE, ultimately improving clinical outcomes.

## Data Availability

The raw data supporting the conclusions of this article will be made available by the authors, without undue reservation.
