# Supplementary material for: Portal vein hemodynamics measured by 4D flow MRI in predicting minimal hepatic encephalopathy in chronic hepatic schistosomiasis patients
Source: Front Med (Lausanne). 2025 Oct 16;12:1681210. doi: 10.3389/fmed.2025.1681210 (PMC12571824; doi:10.3389/fmed.2025.1681210)
Supplement: Supplementary file 1 [file Table_1.DOCX]

**Supplementary Methods**

**Number Connection Test-A**

The Number Connection Test-A (NCT-A) is a timed assessment that primarily gauges psychomotor speed and visual-motor tracking. The test presents the participant with a sheet of paper containing the numbers 1 through 25, randomly scattered. The individual's task is to connect the numbers in ascending order as quickly and accurately as possible.

The score is the total time taken to complete the task, with errors being pointed out and corrected by the administrator during the test, which contributes to the overall time. A longer completion time can indicate deficits in processing speed, visual scanning abilities, and the ability to seamlessly integrate visual information with motor output. The NCT-A is often used as a screening tool for cognitive impairment and to monitor changes in cognitive function over time.

**Digit Symbol Test**

The Digit Symbol Test (DST), also known as the Digit Symbol Substitution Test, is a more complex measure that evaluates a broader range of cognitive functions. This timed test provides the participant with a key that pairs specific digits (1 through 9) with unique symbols. Below the key are rows of digits, and the individual must write the corresponding symbol for each digit as quickly as possible within a set time limit of 90 seconds.

The DST assesses several cognitive domains simultaneously, including processing speed (the ability to quickly perceive and respond to visual information); working memory (holding the digit-symbol pairings in mind while completing the task); attention and concentration (sustaining focus on the task and ignoring distractions); visuomotor coordination (the ability to translate visual information into a precise motor response) ; learning and memory (with practice, individuals may learn some of the digit-symbol associations, which can improve their performance).

The score on the DST is the number of correctly drawn symbols within the time limit. A lower score can be indicative of a wide range of cognitive deficits and is sensitive to the effects of aging, neurological conditions, and psychiatric disorders.

**Supplementary Table 1.** Result of the the multivariable logistic regression analysis.

| Coefficients | Odds ratios | 95% CI | t | P-value |
| --- | --- | --- | --- | --- |
| (Intercept) | 1.91 | 0.83-4.38 | 1.52 | 0.131 |
| Gender | 1.08 | 0.94-1.25 | 1.06 | 0.289 |
| Age | 1.00 | 1.00-1.01 | 0.53 | 0.594 |
| Aspartate aminotransferase | 1.00 | 1.00-1.01 | 2.35 | 0.021 |
| Total bilirubin | 1.01 | 1.00-1.01 | 2.10 | 0.038 |
| Portal peak velocity | 0.98 | 0.96-1.00 | -1.88 | 0.062 |
| Portal mean velocity | 0.95 | 0.92-0.97 | -4.85 | <0.001 |
| Portal area | 1.38 | 1.10-1.73 | 2.75 | 0.007 |
| Left branch flow | 1.01 | 0.97-1.06 | 0.65 | 0.520 |

**Supplementary Table 2.** The clinical and laboratory characteristics and portal vein hemodynamic parameters in MHE and non-MHE cases

| Parameters | MHE(N=52) | non-MHE(N=66) |
| --- | --- | --- |
| Gender |  |  |
| Female | 19 (36.5%) | 29 (43.9%) |
| Male | 33 (63.5%) | 37 (56.1%) |
| Age (y) |  |  |
| Mean (SD) | 68 (8.5) | 67 (11.1) |
| Median [Min, Max] | 70 [47, 84] | 68 [41, 84] |
| Education level |  |  |
| Above primary school | 4 (7.7%) | 6 (9.1%) |
| Below primary school | 48 (92.3%) | 60 (90.9%) |
| NCT-A |  |  |
| Mean (SD) | 66 (11.4) | 37 (8.4) |
| Median [Min, Max] | 64 [51, 96] | 38 [6, 51] |
| DST |  |  |
| Mean (SD) | 28 (8.1) | 45 (14.5) |
| Median [Min, Max] | 31 [10, 39] | 45 [14, 88] |
| AST (U/L) |  |  |
| Mean (SD) | 46 (27.8) | 33 (18.8) |
| Median [Min, Max] | 45 [3, 99] | 30 [10, 107] |
| ALT (U/L) |  |  |
| Mean (SD) | 54 (24.3) | 49 (27.3) |
| Median [Min, Max] | 55 [2, 98] | 43 [8, 155] |
| TB (μmol/L) |  |  |
| Mean (SD) | 32.2 (12.2) | 26.7 (10.7) |
| Median [Min, Max] | 30.7 [9.8, 49.0] | 25.2 [2.0, 46.9] |
| ALB (g/L) |  |  |
| Mean (SD) | 30 (5.3) | 29 (5.5) |
| Median [Min, Max] | 30 [23, 45] | 30 [20, 44] |
| PT (s) |  |  |
| Mean (SD) | 13.6 (3.26) | 12.8 (3.30) |
| Median [Min, Max] | 13.4 [11.6, 15.4] | 12.7 [11.9, 15.3] |
| INR |  |  |
| Mean (SD) | 1.23 (0.13) | 1.24 (0.13) |
| Median [Min, Max] | 1.28 [0.95, 1.45] | 1.21 [0.81, 1.40] |
| PLT (10^9^/L) |  |  |
| Mean (SD) | 95 (58.6) | 111 (53.4) |
| Median [Min, Max] | 69 [59, 279] | 82 [61, 271] |
| Portal peak velocity (cm/s) |  |  |
| Mean (SD) | 17.6 (2.48) | 20.7 (4.46) |
| Median [Min, Max] | 16.9 [13.4, 23.4] | 20.4 [15.0, 29.5] |
| Portal mean velocity (cm/s) |  |  |
| Mean (SD) | 11.6 (1.56) | 15.4 (3.45) |
| Median [Min, Max] | 11.9 [8.7, 15.0] | 15.1 [8.9, 22.3] |
| Portal flow (mL/s) |  |  |
| Mean (SD) | 13.5 (4.31) | 12.7 (5.42) |
| Median [Min, Max] | 12.7 [5.4, 21.0] | 12.4 [4.1, 22.1] |
| Portal area (cm^2^) |  |  |
| Mean (SD) | 1.15 (0.29) | 0.85 (0.32) |
| Median [Min, Max] | 1.14 [0.63, 1.64] | 0.89 [0.41, 1.39] |
| Left branch peak velocity (cm/s) |  |  |
| Mean (SD) | 12.0 (4.05) | 12.6 (3.29) |
| Median [Min, Max] | 11.8 [4.8, 18.3] | 12.8 [5.9, 18.2] |
| Left branch mean velocity (cm/s) |  |  |
| Mean (SD) | 7.9 (3.07) | 8.1 (2.83) |
| Median [Min, Max] | 8.9 [2.9, 12.7] | 8.4 [3.9, 13.5] |
| Left branch flow (mL/s) |  |  |
| Mean (SD) | 3.1 (1.43) | 2.5 (1.94) |
| Median [Min, Max] | 3.2 [0.3, 8.6] | 2.5 [0.2, 13.0] |
| Left branch area (cm^2^) |  |  |
| Mean (SD) | 0.38 (0.23) | 0.33 (0.17) |
| Median [Min, Max] | 0.36 [0.08, 1.48] | 0.33 [0.06, 0.78] |
| Right branch peak velocity (cm/s) |  |  |
| Mean (SD) | 15.1 (1.65) | 15.3 (3.33) |
| Median [Min, Max] | 15.1 [9.1, 18.1] | 15.3 [10.7, 22.5] |
| Right branch mean velocity (cm/s) |  |  |
| Mean (SD) | 9.6 (1.39) | 9.8 (3.73) |
| Median [Min, Max] | 9.8 [3.7, 11.7] | 9.7 [4.5, 16.0] |
| Right branch flow (mL/s) |  |  |
| Mean (SD) | 7.0 (2.65) | 6.2 (2.98) |
| Median [Min, Max] | 7.2 [3.1, 11.2] | 6.3 [0.8, 14.8] |
| Right branch area (cm^2^) |  |  |
| Mean (SD) | 0.67 (0.27) | 0.67 (0.38) |
| Median [Min, Max] | 0.60 [0.25, 1.13] | 0.59 [0.20, 1.27] |

ALB, albumin; ALT, alanine aminotransferase; AST, aspartate aminotransferase; DST, digit symbol test; INR, international normalized ratio; MHE, minimal hepatic encephalopathy; NCT-A, number connection test-A; PLT, platelet count; PT, prothrombin time; TB, total bilirubin
